# Management of flying insects on expressways through an academic-industrial collaboration: evaluation of the effect of light wavelengths and meteorological factors on insect attraction

**DOI:** 10.1186/s40851-020-00163-7

**Published:** 2020-11-26

**Authors:** Masahiro Komatsu, Keigo Kurihara, Susumu Saito, Mana Domae, Naoki Masuya, Yuta Shimura, Shunichiro Kajiyama, Yuna Kanda, Kouki Sugizaki, Kouji Ebina, Osamu Ikeda, Yudai Moriwaki, Naohiro Atsumi, Katsuyoshi Abe, Tadashi Maruyama, Satoshi Watanabe, Hiroshi Nishino

**Affiliations:** 1 Technology Planning Section, Hokkaido Regional Head Office, East Nippon Expressway Co., Ltd., 12-30, Oyachinishi 5-chome, Atsubetsu-ku, Sapporo, 004-0042 Japan; 2 Nexco-Engineering Hokkaido Co., Ltd., 3-20, 5-Jyo 4-chome, Higashi, Sapporo, 003-0005 Japan; 3 Research Institute for Electronic Science, Hokkaido University, Sapporo, 060-0812 Japan; 4 Laboratory of Biology, Hokkaido University of Education, Sapporo Campus, Sapporo, 002-8502 Japan

**Keywords:** Gypsy moth, Oak silkmoth, Chafer, Fluorescent light, Light emitting diode (LED), Light trap, Meteorological factors, Subarctic region

## Abstract

Insect outbreaks often occur in the absence of natural enemies and in the presence of excess suitable host materials. Outbreaks of gypsy moths are especially problematic in remote areas located in high-latitude regions in Japan because the majority of adults emerge during the short summer season and initiate synchronous mass flight toward artificial lights. The aggregation of moths in public facilities not only is an annoyance to visitors but also permits the establishment of new populations the following year. The aim of this study was to establish a method to reduce the numbers of large moths that are attracted to lights in the rest areas of expressways in Hokkaido based on the results of research on their behavioral ecology and physiology. First, we conducted extensive insect surveys using light traps that emit light at different wavelengths; the traps were set along the expressways in the summers of 2014–2018. The insects attracted to the light were roughly classified into those showing a preference for broadband light wavelengths (from UV-A to green) and short light wavelengths (from UV-A to blue). The former included aquatic insects and winged ants, and the latter included moths and beetles. Next, we analyzed correlations between moth emergence and daily meteorological data. When gypsy moths were abundant during an outbreak, the daily catch of gypsy moths was positively correlated with the highest ambient temperature on the catch day but not with the visibility range, wind speed, or moon phase. In contrast, the daily catch of oak silkmoths did not correlate with any of these parameters. Our results provide guidance for the management of forest insects inhabiting cool-temperate to subarctic regions based on light wavelengths with reference to weather variables.

**Supplementary Information:**

The online version contains supplementary material available at 10.1186/s40851-020-00163-7.

## Introduction

Hokkaido, the northernmost island of Japan, has rich biodiversity due to abundant natural resources. Since Hokkaido has no rainy season and has low humidity throughout the year, unlike the main island of Japan (Honshu), it is a popular destination for tourists. More than 50 million tourists (10 times higher than its resident population) visit Hokkaido each year, and the number of people visiting Hokkaido has been gradually increasing. Hokkaido has a long history of periodic insect outbreaks dating back 150 years; records include a locust (*Locusta migratoria*) plague, a tussock moth larvae plague [35], and gypsy moth outbreaks [34]. In the 1990s, oak silkmoths were added to the list of insects causing outbreaks in Hokkaido [30].

**Fig. 1:**
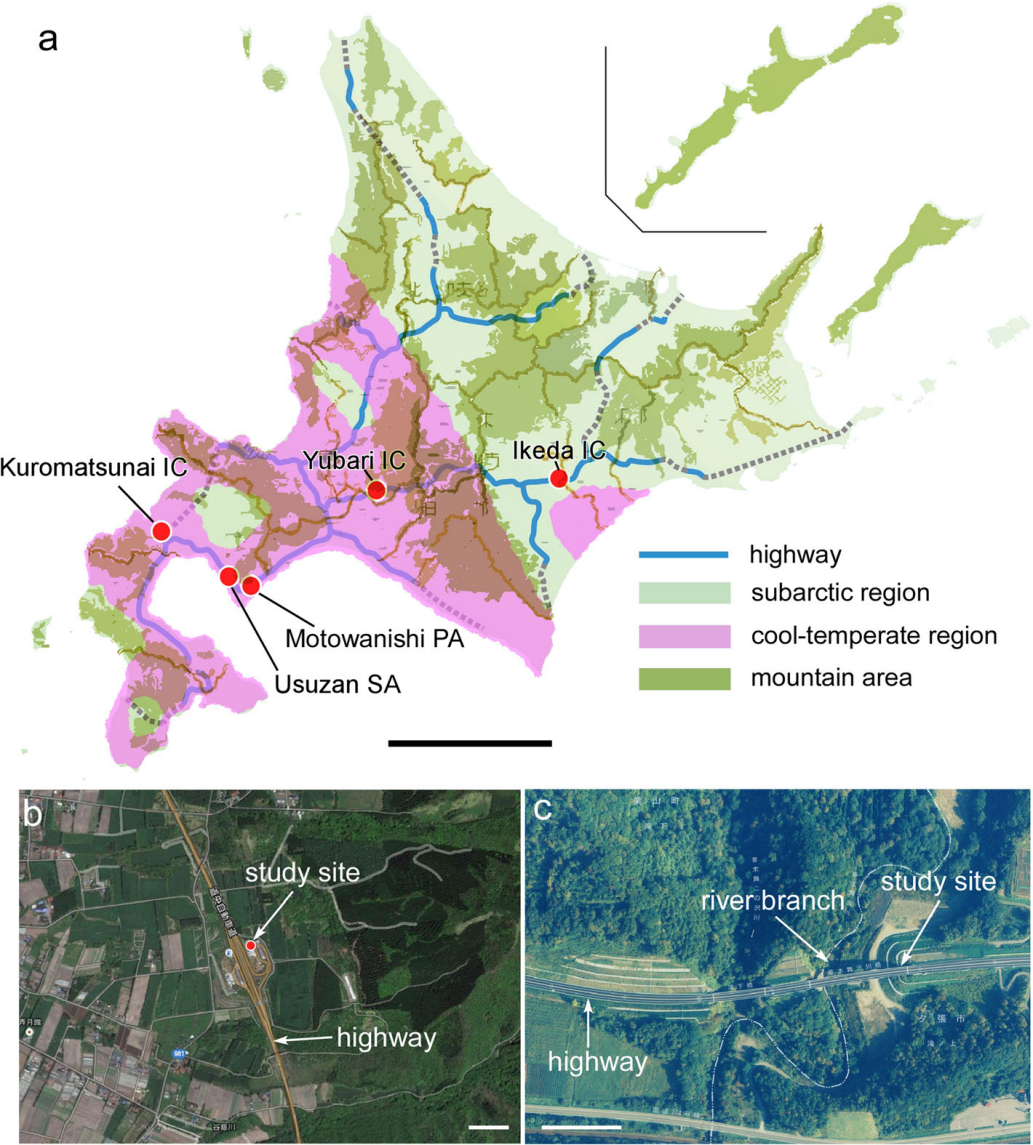
Study sites. Map of Hokkaido (**a**) showing the moth-abundant sites along the expressways managed by E-NEXCO (blue) overlaid on cool-temperate region (magenta) and subarctic region (green) layers. Mountain areas are shown in dark green. Field experiments in 2014 and 2015 were conducted in the back area of the Mt. Usu (Usuzan) rest area (**a**, **b**) and those in 2017 and 2018 were conducted on a hill on flat ground beneath an elevated expressway close to the Yubari Interchange (**a**, **c**). Both study sites had few commercial lights and were surrounded by forests (**b**, **c**). Scale bars = 100 km in a; 200 m in **b** and **c**

Such insect outbreaks are closely related to geographical features and climatic factors [61]. First, forests occupy 66% of the land area of Hokkaido, the largest percentage in Japan (Fig. 1a), and the forests supply rich resources for forest insects. Second, due to the cool-temperate and subarctic climate of Hokkaido (Fig. 1a), most larval insects develop to adulthood within the short summer season, which is suitable for reproduction [57]. Third, natural forests are now being replaced by planted forests due to the afforestation of larches (*Larix leptolepis*) and birches (*Betula maximowicziana*), potentially promoting outbreaks of some species of insects [42].

**Fig. 2:**
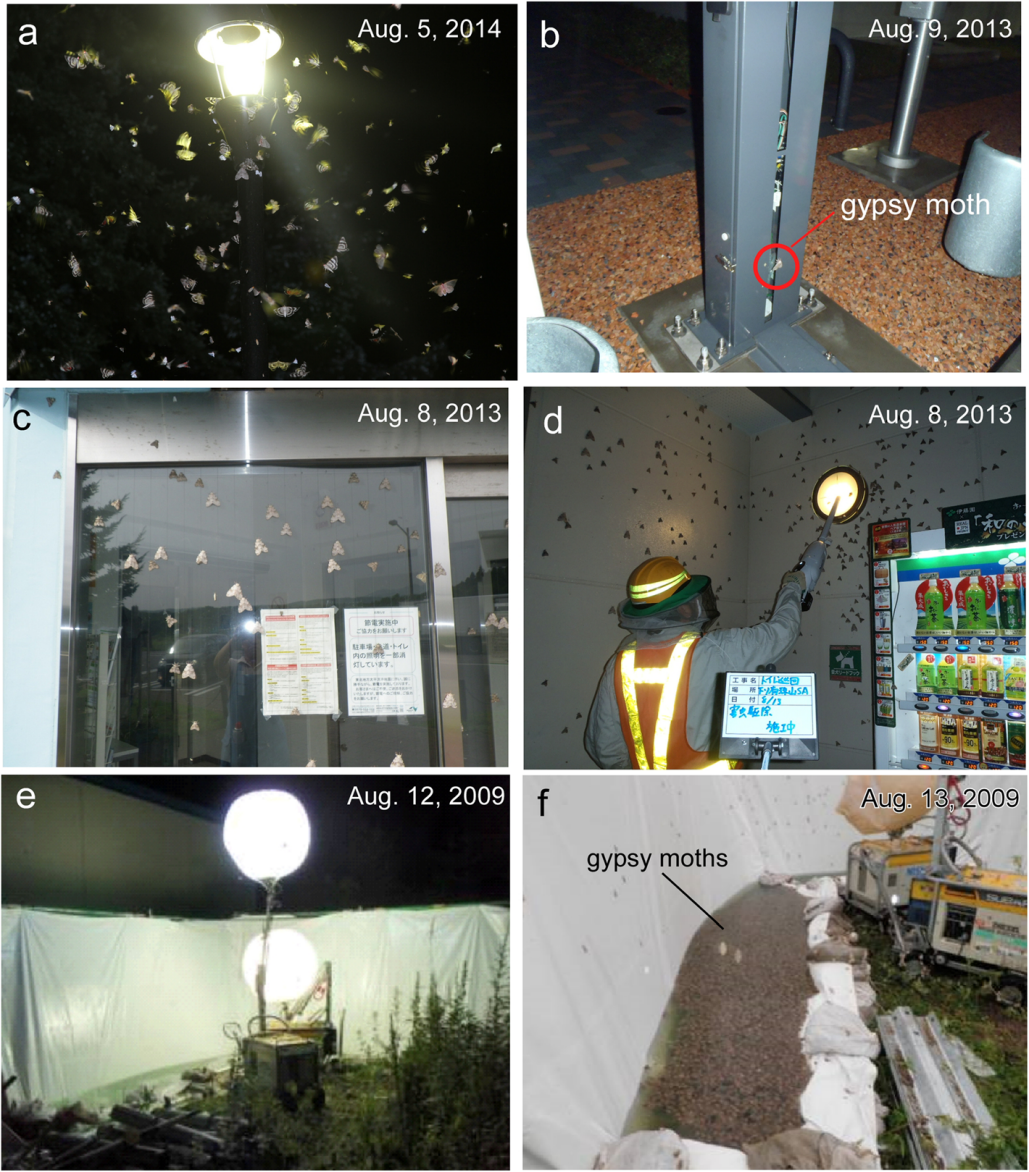
Outbreaks of gypsy moths and sampling using light traps on expressways. Macromoths, such as gypsy moths and oak silkmoths, were lured to commercial lights in August (**a**), which coincided with the peak time of tourism. Errors occasionally occur in the Electronic Toll Collection (ETC) System due to sensor jamming by moths (**b**). Photograph taken at the Kuromatsunai Interchange showing female gypsy moths aggregated on the window of a shop (**c**). Another photograph taken at the Motowanishi Parking Area showing the manpower needed to remove gypsy moths on the walls of toilet rooms (**d**). A high-power balloon light set in the back area of the Ikeda Interchange trapped more than 20,000 gypsy moths in only one night during the outbreak in 2009 in Tokachi district (**e**, **f**). See Fig. 1a for locations of (**c**)-(**e**). The photograph (**a**) taken at the base of Mt. Daisetsu in central Hokkaido was courtesy of Dr. H.J. Pflüger

Gypsy moths (*Lymantria dispar*) are ranked as the world’s worst invasive alien species due to their euryphagous habit of feeding on 100–300 species of plants, including larches [47]. Outbreaks of gypsy moths occur approximately every 11 years in Hokkaido [34, 42]. The Asian gypsy moth, *L. dispar japonica*, is endemic in Japan, and three related species have been reported in Hokkaido [3–5]. Unlike female European gypsy moths, *Lymantria dispar dispar*, which are not flight-capable [27, 66], all female *Lymantria* species in Hokkaido are flight-capable (Fig. 2a) [36]. *Lymantria* species are univoltine, with the larval stage occurring in May to July and the adult stage occurring in mid-July to early September [57]. Eggs undergo diapause in winter, with development stopping immediately before hatching [4, 5].

The Japanese oak silkmoth, *Saturnia japonica* (Saturniidae), is one of the largest moths in Hokkaido. The oak silkmoth has a life cycle and feeding habit similar to those of *L. dispar* [50], but the adults emerge later, around September and October, in Hokkaido [30]. Outbreaks of oak silkmoths tend to occur locally and are smaller in scale than those of gypsy moths [30].

The gypsy moth and oak silkmoth are considered macromoths (or macrolepidoptera), with wingspans reaching 63 mm in female *L. dispar* and 110 mm in female *S. japonica.* Both species of moths do not feed in the adult stage and do not engage in pollination, unlike pyralid moths or sphingid moths [44].

In addition to the agricultural impact of insect outbreaks, a relatively underestimated and even overlooked problem is the aesthetically negative impact of insect outbreaks on public facilities [68]. Most of the expressways in Hokkaido are toll roads managed by East Nippon Expressway Company Limited (E-NEXCO), and the expressways pass through rural areas with mountains and rivers (Fig. 1a). When outbreaks occur, substantial numbers of gypsy moths fly toward the lights in the rest areas of the expressways and lay their eggs on artificial objects [43, 59]. Errors sometimes occur in the Electronic Toll Collection (ETC) System due to sensor jamming from insects (Fig. 2b). Moths that aggregate on the windows of expressway shops (Fig. 2c) and on the walls of toilet rooms (Fig. 2d) frighten tourists. Moreover, some people experience allergic reactions to the scales of moths [72].

A few environmentally friendly control methods targeting *L. dispar* have been used in North America. The female-emitted sex pheromone of *L. dispar* was synthesized and is commercially sold as “disparlure”, which interrupts mating [28]. However, the sex pheromone lures males but not females, which produce offspring. A biopesticide (*Gypchek*) produced by the U.S. Forest Service is currently on the market [55], but it is effective only when used during the early larval stage, preferably the first larval instar stage [55]. Therefore, this would not be effective in achieving our goal of eliminating postcopulated females, the primary target of our study, from rest areas. Excessive use of agrichemicals should be avoided in rest areas, which are public spaces where tourists frequent.

From the viewpoint of prevention, the long-term population dynamics associated with environmental factors among forest defoliators, such as *Lymantria*, have been a major issue in forest ecology [27, 34, 54]. Given that outbreaks occur locally in Hokkaido due to the complex landscape [34], populations and meteorological parameters affecting adult emergence should be determined locally.

Light trapping is a classic yet highly effective method to evaluate local population dynamics of insects [6, 23, 33, 46, 54]. Light-emitting diodes (LEDs) that radiate short light wavelengths manipulate insect behavior because most insects utilize light of particular wavelengths as navigational cues in phototaxis [8, 71]. Light traps have been used for sampling agricultural pest insects [51, 60]. Since a substantial number of gypsy moths were captured in only one night by using a light trap in central Hokkaido in 2009 (Fig. 2e, f), we decided to use light traps to sample flying insects and hopefully reducing their populations. Collaboration between E-NEXCO, which owns private yards suitable for field surveys along expressways, and researchers with contemporary knowledge about the behavioral ecology and physiology of insects would be beneficial for achieving environmentally friendly pest management.

There were two objectives for the light trap experiments conducted in this study. Since older lights, such as incandescent lights, are now rapidly being replaced with ultraviolet (UV)-free LED lights on expressways, our first priority was to identify major insect species that congregate at expressway lights and to estimate how the density and diversity of insect species attracted to visible-light wavelengths are affected by UV light. Second, to forecast the emergence of adult moths, determining the correlations between adult emergence and meteorological factors is important. In contrast to abundant studies about monthly and annual moth catches and long-term weather variables [15, 22, 62], there have been very few systematic studies in which correlations between daily moth catch and weather variables were investigated [33, 37]. Since each expressway has a real-time weather monitoring system, we used the meteorological data from the systems near the light traps for analysis.

In this study, we conducted an extensive survey of insects by using light traps over a four-year period in Hokkaido. Our light traps, which were deployed alongside expressways, enabled the sampling of various insect species in summer. Light-source orientation in some species, such as *L. dispar,* can be predicted on the basis of weather variables and can be controlled by using LEDs that emit light of specific wavelengths.

## Materials and methods

### Specimens

All of the trapped insects underwent species classification, though we paid special attention to macromoths, which are regarded as nuisance insects by visitors.

### Experimental periods

Gypsy moth outbreaks in Hokkaido started in 2012, peaked in 2013 (see https://www.youtube.com/watch?v=BKeW-MtlZIs), and declined in 2014. Following the spread of nuclear polyhedrosis virus (NPV), commonly known as baculovirus, among larvae and pupae in 2014, outbreaks ceased from 2015 to 2019, a period referred to as the innocuous or endemic phase [27]. Hence, we were not able to collect sufficient numbers of gypsy moths at the Mt. Usu rest area in 2015 and 2016. In the early spring of 2017, we spotted gypsy moth egg masses on the a of an elevated expressway close to Yubari Interchange, and we conducted field experiments in the summers of 2017 and 2018 (Additional file 1).

Light trap surveys were conducted from mid-July to the end of September in 2014, 2015, 2017, and 2018. In this study, we paid special attention to the relationships between insects and weather parameters in 2014 and 2018, years for which daily counts of trapped insects were available.

### Study sites

During an intense outbreak of gypsy moths that occurred in central Hokkaido (Tokachi district) in 2009, E-NEXCO set up a balloon-light trap for one night in the back area of the Ikeda Interchange (42°59′45.0″N 143°26′15.7″E, elevation: 23 m) (Figs. 1a and 2e,f). Since then, East Nippon Expressway Company Limited has conducted preinspections for gypsy moth egg masses and larvae from April to June each year. Accordingly, two sites alongside expressways were selected because they were moth-abundant sites. One was located on the coastline, and the other was located inland. The former was the back area of the Mt. Usu (Usuazan) rest area (Fig. 1a) along the outbound line (42°28′02.3“N 140° 54’31.6”E, elevation: 121 m), located approximately 200 m from commercial lighting (Fig. 1b). The latter was a hill on flat ground (42°54′55.8″N 141°58′02.0″E, elevation: 130 m) located beneath an elevated expressway (Additional file 1) near the Yubari Interchange (42°55′26.3″N 142°02′10.6″, elevation: 121 m), approximately 100 m from a branch of Yubari River (Fig. 1c). The site had no commercial light sources for 300 m in all directions and was surrounded by forest (Fig. 1c).

### Configurations of light traps

**Fig. 3:**
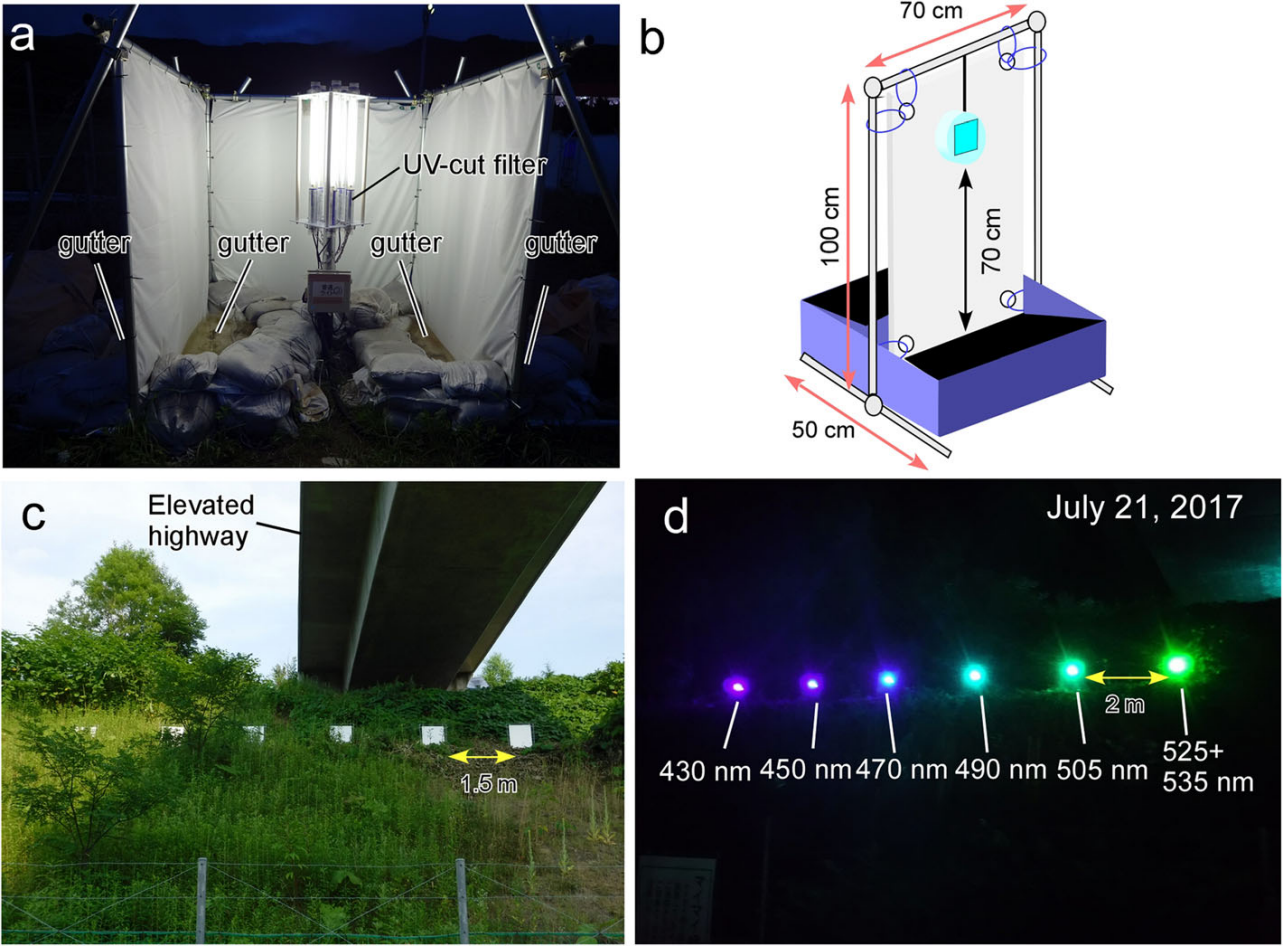
Configurations of light traps used in the field experiments. **a** “Light tower” trap used in 2014 and 2015. See Additional file 2 for details. **b** Flight-interception LED traps used in 2017 and 2018. See additional file 3 for details. Six flight-interception LED traps that emitted light with different light wavelengths (**d**) were set on a hill on flat ground beneath an elevated expressway (**c**). The intensity of total illumination for each LED module was adjusted to approximately 1500 lm (**d**)

In 2014 and 2015, we used “light tower” traps [6, 12]. Each trap consisted of a U-shaped white tarpaulin (thickness: 3 mm) with an array of fluorescent lights placed vertically in the middle (Fig. 3a, Additional file 2). Traps were set 2 m apart. Basins for catching insects that dropped from the tarpaulin were made inside and outside the base of the tarpaulin and filled with water (Fig. 3a). To ensure a nonadherent surface, a kitchen detergent was applied to both sides of the tarpaulin. All of the insects in the basins were collected manually from each trap the next morning, and their wet weights were subsequently measured.

To reduce manpower, a flight-interception LED trap, also referred to as an “automatic funnel trap” [6, 13], was used in 2017 and 2018 (Additional file 3). Each LED module was fixed in the center of a corrugated plastic board (thickness: 5 mm) that was positioned vertically against the collection box (Fig. 3b). A funnel was placed on the top of the collection box to prevent trapped insects from escaping (Fig. 3b). Traps were placed 1.5 m apart (Fig. 3c) and the distance between lights was set at 2 m (Fig. 3d). Electricity was provided by the main building or mobile batteries via long extension power cables. In all the experimental periods, the lights were turned on and off with a timer.

We rotated the location of the traps clockwise at each site on successive collection nights to ensure at least one night at each position. Unless otherwise stated, all specimens collected were removed from the traps after each collection night and taken to the laboratory for sorting and subsequent species identification.

The emission power spectra of each light and its illuminance (lx) were measured at 80 cm from the light using a spectroradiometer (CL-500A, Konica Minolta, Japan) and are presented as the average of 10 consecutive measurements. The emission intensity of the UV light range (290–390 nm) was measured by a UV intensity meter (UVK-40 M, JEFCOM, Japan). The detailed configurations of the light traps for each year are as follows.

***2014*** To evaluate the effects of commercial lights that emit broadband wavelengths on insect attraction, four “old-type” of fluorescent tubes were deployed in the back area of the Mt. Usu rest area (See Additional file 2 for specifications of lights.). The lights were 1) neutral white (Ra: 70) fluorescent lights (φ32.5 mm, 1170 lm × 5 = 5850 lm, FL20SS・N/18, Toshiba Lighting & Technology Corporation); 2) LED lights (φ32.5 mm, T8T-S562F50, Inaba Denki Sangyo Co., Ltd.); 3) UV-free lights (Ra: 90) (φ32.5 mm, 850 lm × 7 = 5950 lm, FL20S・N-SDL・NU, Toshiba Lighting & Technology Corporation), and 4) black lights (φ32.5 mm, 12 tubes, FL20S・BLB, Toshiba Lighting & Technology Corporation). To eliminate UV light, each of the lights was entirely covered with a polycarbonate transparent filter that blocks UV light (PDW-VF20, JEFCOM, CO., LTD., thickness: 2 mm). The intensity of total illumination for each trap was adjusted to approximately 6000 lm except for the black light (Additional file 2). The lights were turned on at 18:00 and turned off at 6:00 the following day.

***2015*** To evaluate the effects of colors of light, we used the following colored fluorescent tubes with UV-free filters (PDW-VF20, JEFCOM, Japan): 1) blue fluorescent tubes (FLR40S.EB/M. A, Toshiba Lighting & Technology Corporation), 2) turquoise-blue fluorescent tubes (FL20S・BW, Toshiba Lighting & Technology Corporation), 3) green fluorescent tubes (FL20S・G, Toshiba Lighting & Technology Corporation), and 4) neutral white fluorescent tubes (FL20SS・N/18, Toshiba Lighting & Technology Corporation). The tubes were deployed in the back area of the Mt. Usu rest area (See Additional file 2 for light specifications). For each trap, the intensity of illumination was adjusted to approximately 5000 lm (Additional file 2). The lights were turned on at 18:00 and turned off at 6:00 on the following day.

***2017*** **and** ***2018*** To evaluate the effect of UV light on insect attraction, LED modules that emit light from UV-A to visible-light wavelengths were deployed in the experimental site near the Yubari Interchange (see Additional file 3). Each LED module consisted of multiple spherical LED chips emitting light with wavelengths of 365, 375, 405, 430, 450, 470, 490, 505, 525, and/or 535 nm (LED-ON, Japan); the modules were set into LED bulb sockets arrayed on a specialized circuit board (Spectrolight SPL-25-CC, LED-ON, Japan), which was connected via an AC adapter to the outlet (AC100V) of a generator. The intensity of the total illumination for each LED module was adjusted to approximately 1500 lm (Fig. 3d). The light source was covered with transparent UV light-transmitting plastic for rain protection (Fig. 3b). The LED modules were turned on at 18:00 and turned off at 2:00 on the following day.

### Measurements of meteorological factors

On each night of collection, ambient temperature, visibility range, and wind speed were automatically recorded every 5 minutes by data logging systems on the expressway near the light traps. During the periods when light traps were used, the overnight temperature ranged from 5.9 °C to 33.8 °C, and the wind speed ranged from 0 m to 22.2 m/s. The moon score was calculated as the summation of the weighted score of the moon size and the meridian passage of the moon (highest possible score: 10 points). The time of 21:00 h was regarded as the postsunset time when moths exhibit the highest degree of flight activity. Five points represented a full moon, and zero points represented a new moon. Another five points were assigned when the meridian passage of the moon occurred at 21:00, and zero points were assigned when the meridian passage of the moon occurred at 9:00.

### Insect sorting and identification

In 2014, the insects trapped at the Mt. Usu rest area were mostly *Lymantria dispar*, and further species identification was therefore omitted. The mass weight of the moths was measured immediately after one collection night. In 2015–2018, the insects trapped in each trap were photographed and lightly dried in an oven (50 °C). We then transferred the specimens to plastic dishes and observed them with the naked eye. We sorted the insects by taxonomic order with guidance from the River Environmental Database supplied by the Ministry of Land, Infrastructure, Transport and Tourism of Japan (http://www.nilim.go.jp/lab/fbg/ksnkankyo/mizukokuweb/system/seibutsuListfile.htm). The gypsy moth *Lymantria dispar japonica*, which inhabits Honshu and Hokkaido, and the closely related species *Lymantria umbrosa,* which inhabits only Hokkaido, have a very similar external appearance and are distinguishable only by their genitalia [4]. Due to the physical damage to gypsy moths caused by the traps, we regarded the samples as gypsy moths. For the same reason, closely related species of oak silkmoths, *Antheraea yamamai ussuriensis* and *Saturnia jonasii fallax* Jordan, were regarded as oak silkmoths. For small insects for which species identification was difficult (e.g., flies, mosquitoes, and winged ants), the order, family or genus to which they belonged was identified.

### Data evaluation

Using the nonparametric Kruskal-Wallis H test, we examined the data to determine if the distribution was normal. Subsequently, multiple comparisons were made by the Steel-Dwass test using add-ins in Excel (Excel statistics ver. 7.0, Esumi, Japan). This examination revealed a 95% reliability level. Eight daily meteorological variables were selected as possible meteorological predictors correlated with trap catches: highest temperature, mean temperature, dusk temperature (at 20:00), lowest temperature, moon score (see above), average wind speed from 18:00–24:00, maximum wind speed, and visibility range from 18:00–24:00. The data were subjected to correlation and least squares linear regression analyses [11]. We regarded R^2^ values of 0.2–0.4 as indicating weak correlations and R^2^ values of 0.4–0.7 as indicating moderate correlations.

## Results

### Abundance of flying insects on expressways in Hokkaido

We were able to collect many insect species from multiple orders by using light traps, although obviously the trapped insects do not represent all of the species that inhabit expressways. The insect orders identified were Odonata, Dermaptera, Orthoptera, Hemiptera, Ephemeroptera, Neuroptera, Trichoptera, Lepidoptera, Diptera, Coleoptera, and Hymenoptera (Additional file 4), indicating a rich diversity of entomofauna despite the cool-temperate and subarctic climate (corresponding to ***Df*** by Köppen’s Climate Classification) of Hokkaido (Fig. 1a). Important pollinators, including butterflies, bumblebees, pyralid moths, and sphingid moths, were rarely captured in our traps (Additional file 4). Arctiid moths (*Chionarctia nivea*), which resemble gypsy moths, were specifically trapped in late July (Additional file 4; see also Fig. 5b).

**Fig. 4:**
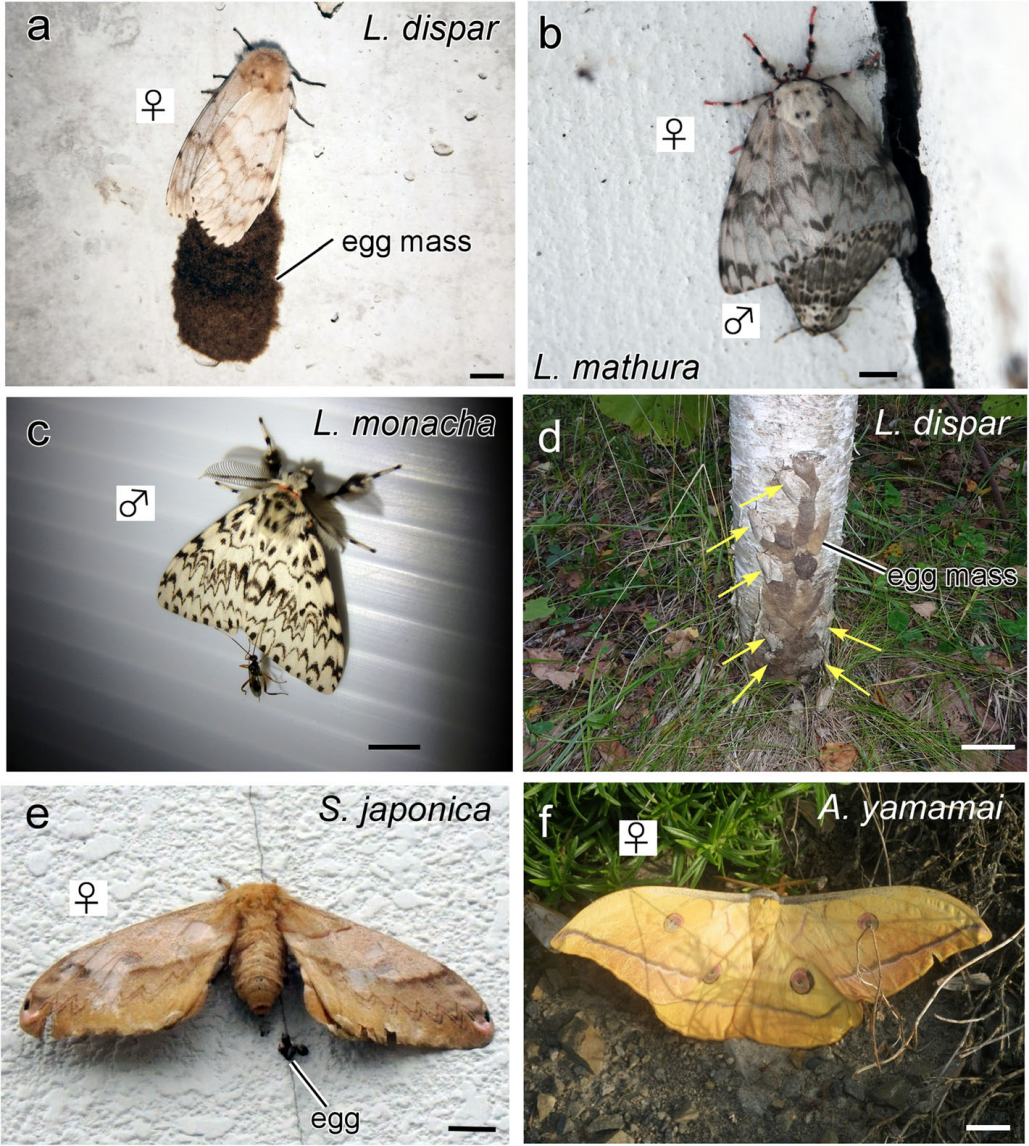
Target moth species to be controlled on expressways include three species of gypsy moths (*Lymantria*), *Lymantria dispar* (**a**), the pink gypsy moth *Lymantria Mathura,* (**b**) and the nun moth *Lymantria monacha* (**c**); and two species of Japanese oak silkmoths (Saturniidae), *Saturnia japonica* (**e**) and *Antheraea yamamai* (**f**). A parasitoid wasp is approaching from the rear of an *L. monacha* moth (**c**). Gypsy moths usually lay egg masses on trunks of white birches (moths indicated by yellow arrows, **d**); thus, artificial objects with a white background are favored as egg-laying sites (**a**). Photographs in (**b**) and (**f**) are courtesy of Dr. H.J. Pflüger. Scale bar = 1 cm

In light of the numbers of insects trapped (Additional file 4) along with reports from visitors to rest areas, the following macromoths were recognized as nuisance insects requiring control (Fig. 4). The primary targets were gypsy moths (superfamily: Noctuidae, family: Erebidae) [4], including *Lymantria dispar japonica* (or *Lymantria dispar umbrosa*) (Fig. 4a) and its related species, the pink gypsy moth, *Lymantria mathura* (Fig. 4b), and the nun moth *Lymantria monacha* (Fig. 4c). The secondary targets were saturniid moths (family: Saturniidae) [30]: Japanese oak silkmoths *Saturnia japonica* (Fig. 4e) and *Antheraea yamamai* (Fig. 4f).

Female gypsy moths fly vigorously at dusk to locate mates and egg-laying sites [36, 39], while Japanese oak silkmoths fly actively throughout the night, similar to other saturniid moths [41]. We spotted egg masses of gypsy moths and oak silkmoths on artificial objects with a white background at the end of September (Fig. 4a,c,e) [43] because their primary egg-laying sites in natural habitats are white birches (Fig. 4d, each moth is indicated by a yellow arrow) [31, 58].

### Effects of visible light wavelengths on insect attraction

**Fig. 5:**
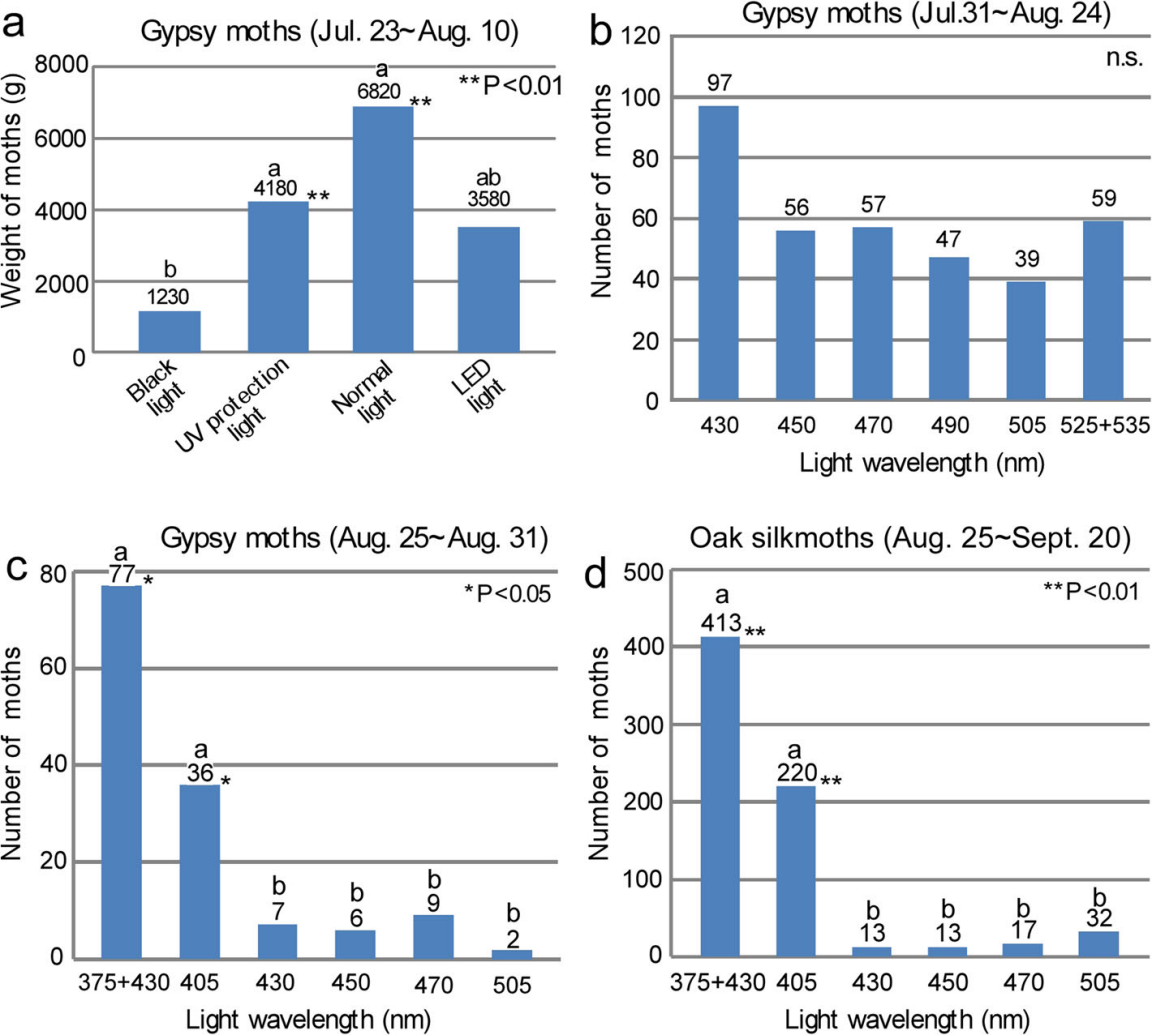
Effects of light wavelengths on gypsy moth and oak moth attraction. **a** Attraction of gypsy moths to four “old-type” fluorescent lights with UV light emission (< 380 nm) eliminated. **b**, **c** Attraction of gypsy moths before (**b**) and after the introduction of a UV LED to an array of LEDs with visible-light wavelengths (**c**). **d** Attraction of Japanese oak silkmoths to UV and visible-light LEDs. The results shown in (**a**) were from experiments at the Mt. Usu rest area in the last year (2014) of the gypsy moth outbreak, and the results shown in (**b**-**d**) were from experiments in Yubari in 2018. Means not sharing the same letter are significantly different (Steel-Dwass test, *P < 0.05, ** *P* < 0.01)

In 2014, we tested the attraction of moths to different kinds of lights that emit broadband light wavelengths; each was covered with a UV-free filter to eliminate light with wavelengths shorter than 380 nm (Fig. 3a; Additional file 2). A black light was used as the negative control, and the UV-free black light attracted very few insects. Among white LED lights, UV-free fluorescent lights, and normal fluorescent lights, the normal fluorescent lights (see Additional file 5a for the power spectra of the lights) attracted approximately twice as many gypsy moths than the other two types of lights (Fig. 5a). The normal fluorescent light had a higher spectral intensity in the range of 400–450 nm than the other two configurations of lights (Additional file 5a).

**Fig. 6:**
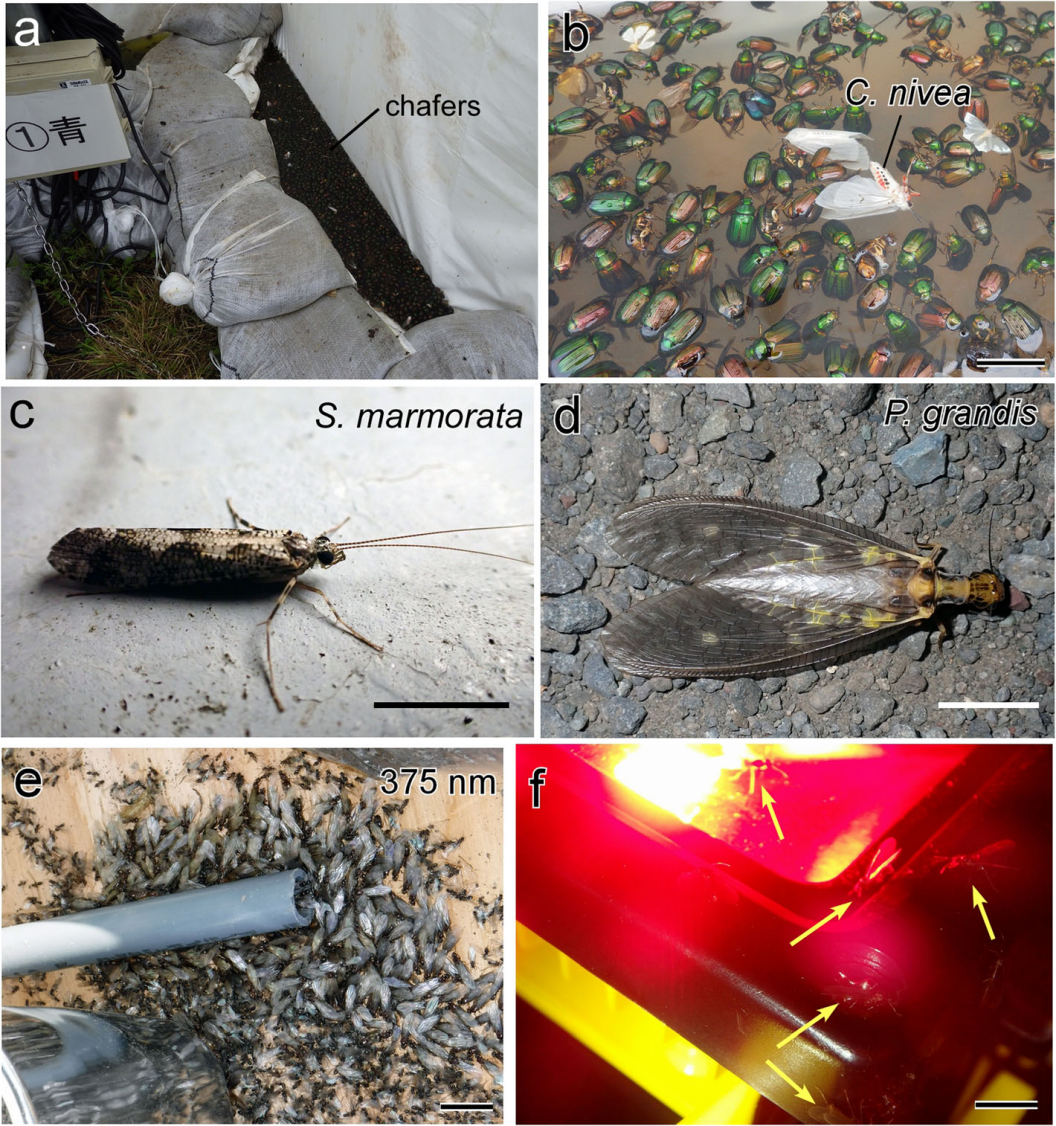
Insects (other than moths) attracted to light that were abundant on expressways. A substantial number of common chafers were attracted to blue light and trapped in the basin (**a**, **b**). Note that two Arctiid moths (*Chionarctia nivea*) were intermixed with the chafers (**b**). Aquatic insects, including caddisflies (*Stenopsyche marmorata*) (**c**) and dobsonflies (*Protohermes grandis*) (**d**), were attracted to any light sources with a sufficient intensity. Unidentified winged ants were attracted not only to the UV LED (**e**) but also to red light (**f**) illuminated on the ground. Scale bars = 2 cm in **b**, **e**, **f**, 1 cm in **c** and **d**

In 2015, gypsy moths were not found at the Mt. Usu rest area, but many chafers (family: Scarabaeidae), including *Mimela testaceipes*, *Mimela costata*, and *Anomala rufocuprea,* were trapped. These chafers are pests that consume a wide range of conifers and hardwoods [30]. On July 28, approximately 2500 chafers were attracted to the blue fluorescent lights (Fig. 6a, b) but not to the turquoise-blue, green, or white fluorescent lights (see Additional file 5b for the power spectra of the lights). The blue light had a narrow peak from 430 to 450 nm (Additional file 5b). These results were consistent with those in 2017 and 2018, during which the largest number of chafers were attracted to blue LEDs that emitted 430 nm or 450 nm at peak intensity (Additional files 5c and 6).

### Effects of UV LEDs on macromoth attraction

To evaluate the behavioral dynamics of gypsy moths in response to light sources without and with UV light, we introduced UV LEDs to an array of visible-light LEDs in the middle of the peak emergence of gypsy moths in 2017. Before the introduction of UV LEDs, gypsy moths tended to be more attracted to LEDs with blue light (430 nm) than those emitting longer wavelengths, although the differences in attraction among the different LED modules were not statistically significant (Fig. 5b). When UV light (375 nm) was introduced at the same study site, approximately 56% of the moths were drawn to the UV LEDs (Fig. 5c). The 405-nm LED lured fewer moths than the 375 + 430 nm LED, but the difference was not statistically significant (Fig. 5c). Oak silkmoths showed strong preferences for LEDs with short wavelengths of 375 nm + 430 nm and 405 nm (Fig. 5d). In the field, we often observed that gypsy moths that entered the path of radiated green light (500 nm) abruptly changed their flight path or dived to the ground several feet from the source [29]; this was in contrast to their behavior of continuous flight in close proximity to UV light sources (Kurihara and Nishino, personal observations).

### Light spectral preference of aquatic insects and ants

The preference for light wavelengths differed among distinct insect orders. Since the study site in 2017 was located near a river branch, adult aquatic insects, such as the caddisfly, *Stenopsyche marmorata* (Fig. 6c), and the dobsonfly, *Protohermes grandis* (Fig. 6d), were trapped from July to mid-August. These insects tended to be attracted not only to UV-A light but also to light with longer wavelengths, such as green light (Additional file 6), suggesting a preference for broadband wavelengths. These tendencies were generally supported by our casual observations in rural areas of Sapporo that caddisflies and dobsonflies are attracted to any kind of light, including sodium lights along rivers on warm summer nights. Winged ants, which were not identified to species, tended to be attracted to any light sources, including those emitting red light (Fig. 6e, f).

### Meteorological factors that affect insect attraction

**Fig. 7:**
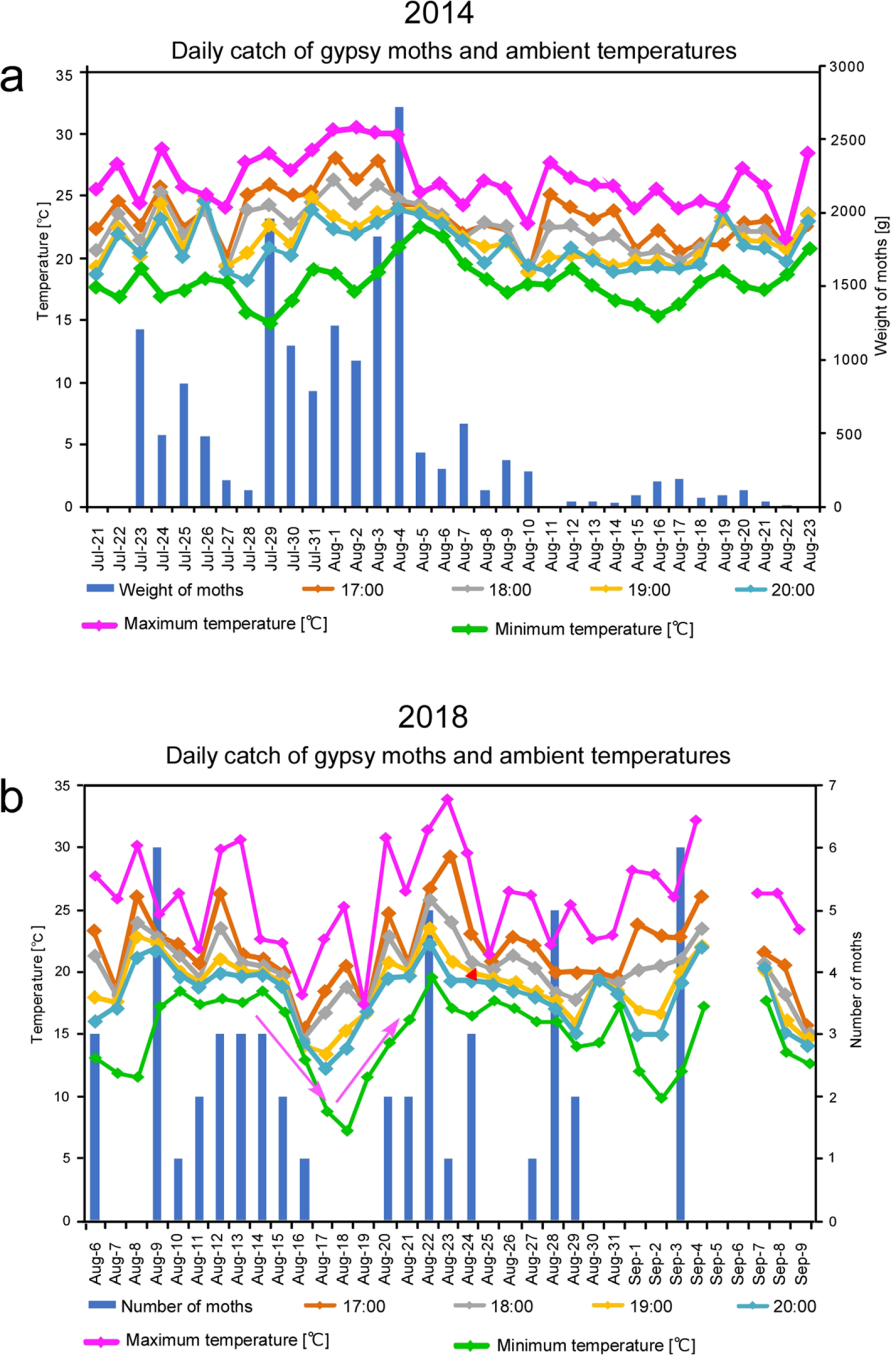
Relationships between daily capture of gypsy moths and temperatures. The total numbers of moths attracted to all light traps set at Mt. Usu 2014 (**a**) and Yubari 2018 (**b**) were plotted

Moth emergence and flight times to light traps are known to be affected by abiotic factors such as photoperiod, moonlight, temperature, wind speed, light trap functioning, and background illumination [7, 45]. The number of insects captured varied greatly among collection dates during the experimental periods (Fig. 7). Determining the correlations between daily catch and immediate weather variables is therefore important for forecasting the emergence of adult gypsy moths.

**Fig. 8:**
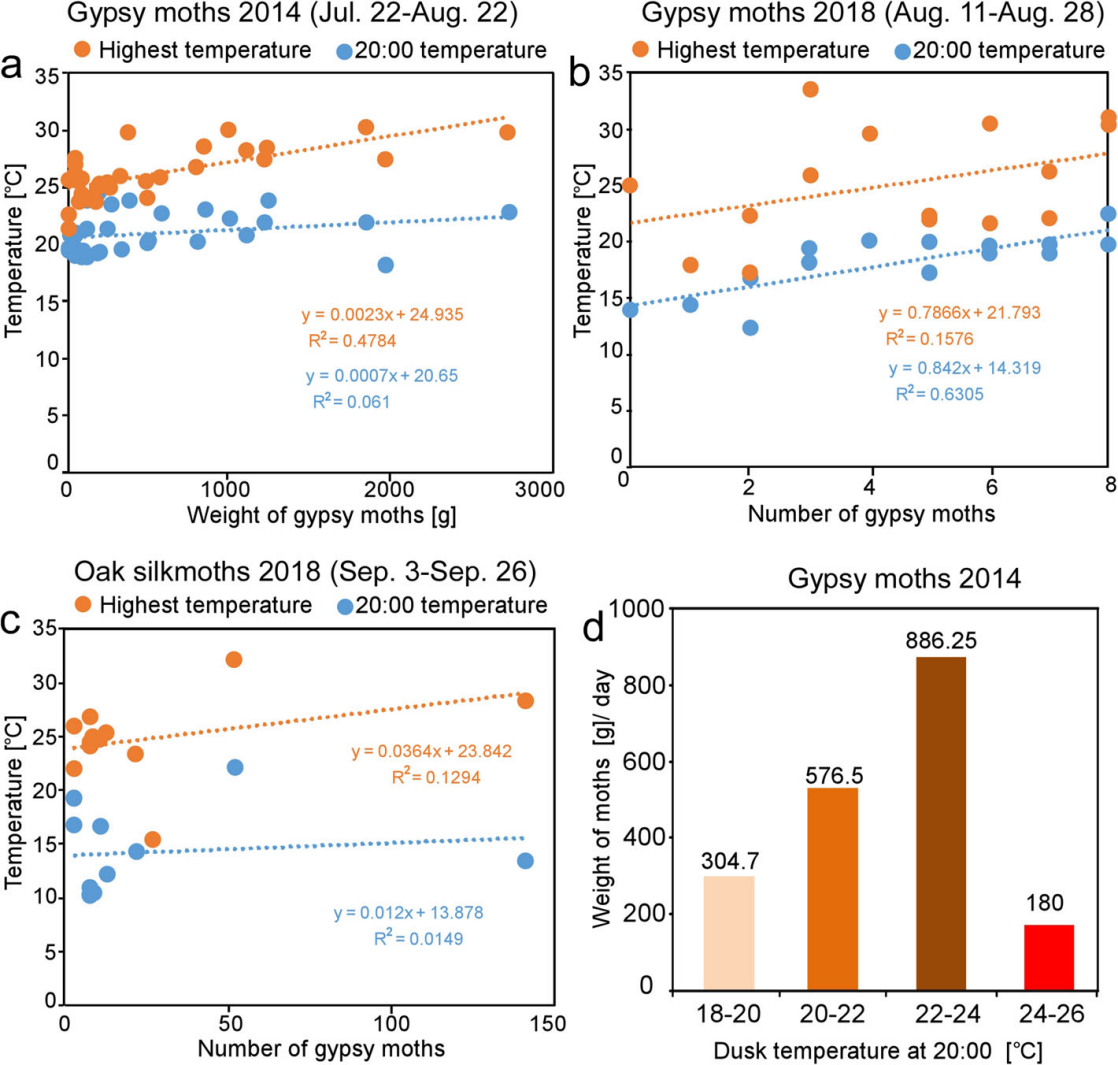
Correlation analyses between daily moth catch and ambient temperatures on each catch day. At the Mt. Usu rest area in 2014 (**a**), the gypsy moth catch was correlated more strongly with the highest temperature than with the dusk temperature, but the opposite correlations were found in Yubari in 2018 (**b**). There were no correlations between oak moth catch and the highest temperature and dusk temperature (**c**). The highest number of gypsy moths was trapped when ambient temperatures ranged from 22 to 24 °C, but the number decreased at temperatures above 24 °C (**d**)

The summer of 2014 corresponded to the last year of the gypsy moth outbreak that began in 2012 in Hokkaido. Among the lowest temperature, highest temperature, and dusk temperature at the Mt. Usu rest area, the highest temperature was positively correlated with gypsy moth catch (R^2^ = 0.478, Fig. 8a,b; Additional file 7). After the maximum number of female gypsy moths (more than 2500 moths) was trapped on August 4, the number of captured moths suddenly declined on August 5 (Fig. 7a). The weak correlation between moth catch and dusk temperature can be explained by the fact that temperatures higher than 24 °C at dusk (20:00) adversely resulted in a lower catch rate of gypsy moths (Fig. 8d), suggesting that gypsy moths have a favorable temperature range (20–24 °C) for flight activity.

In 2017 and 2018, gypsy moth populations were scarce throughout Hokkaido and emerged locally only in the Yubari area (Fig. 7b). As in 2014, the moth catch drastically decreased when the dusk temperature, but not the highest temperature, dropped on August 17–20 (indicated by arrow, Fig. 7b). In fact, the correlation between gypsy moth catch and highest temperature was weak (R^2^ = 0.1576, Fig. 8b), but the correlation between gypsy moth catch and dusk temperature at 20:00 was strong (R^2^ = 0.6305; Fig. 8b). The correlation between gypsy moth catch and wind speed was weakly negative (R^2^ = 0.2186 for average wind speed and R^2^ = 0.3435 for maximum wind speed; Additional file 8d, e). In these years, Japanese oak silkmoths tended to emerge constantly in September, though the number of oak silkmoths was smaller than that of gypsy moths during the outbreak. The oak silkmoth catch at the same study site did not correlate with any of the weather variables (Fig. 8c; Additional file 9).

## Discussion

We have presented fundamental results of field surveys conducted along expressways with the aim of establishing environmentally friendly control methods for flying insects in Hokkaido. Unfortunately, our survey periods coincided with the decline and innocuous phases of gypsy moths [27]. Nevertheless, we were able to identify locations where gypsy moths and oak silkmoths emerge locally and evaluate the effects of light wavelengths and weather parameters on the emergence of these moths. Our data provide guidance for species-specific control of insects in low-temperate to subarctic regions with large-scale emergence under preferable weather conditions [70].

We found that the normal fluorescent light attracted twice as many gypsy moths as the UV-free fluorescent light and a UV-free LED light. This is probably because the spectral intensity of 400–430 nm preferred by nocturnal moths [20, 23, 52] was higher in the normal fluorescent light than in the other lights. When gypsy moths were abundant (e.g., 2014), even UV-free LED lights, which are now mainly used for street lighting, attracted gypsy moths. The numbers of moths attracted to LEDs that emitted distinct visible light wavelengths tended to be larger for shorter wavelengths, but the difference was not statistically significant. Our results are also consistent with the results of studies that showed that lepidopteran insects are more attracted to UV-free LEDs with higher color temperatures (blue) than to those with lower color temperatures (green), but the attraction difference among distinct wavelengths is small [52, 69].

Interestingly, our results showed that when a UV light trap was introduced in an array of visible-light traps at the same study site, approximately 56% of the moths were drawn toward the UV light trap when the intertrap distance was small (1.5 m). This is consistent with the finding that even a small amount of UV radiation triggers behaviors associated with positive phototaxis in insects [8].

Our field surveys using LED traps revealed unique preferences of other insects for particular light wavelengths. For example, surveys conducted at different sites and in different years consistently showed that common chafers in Hokkaido were attracted to blue light at 430–450 nm in the absence of UV light. Therefore, an LED trap that emits this wavelength range might be useful for the specific control of chafers, which are defoliators of conifers and hardwoods in Hokkaido [30]. For other Coleopteran species, the UV-A range has been reported to be more attractive than blue light [25, 32, 38].

Some aquatic insects, such as caddisflies and dobsonflies, showed preferences for broadband light wavelengths, consistent with our casual observations that these insects are attracted to any light, including sodium lights, with sufficient intensity [53]. Red and black light reflection and reflection-polarization signals are known to affect the phototactic behavior of aquatic insects [10, 40]. Therefore, aquatic insects appear to use multiple cues during nocturnal navigation. Since aquatic insects are indicators of water quality and are important for maintaining the ecosystem as a food source for fish [56, 64], we need to consider light intensity to prevent light pollution in an aquatic ecosystem when artificial lights are used along a river. Surprisingly, unidentified winged ants were attracted even to red light, in agreement with the results of a study showing that some ant species are capable of discriminating red from other colors and have behavioral sensitivity to 570–620 nm (orange-red spectrum) [2]. Our findings suggest that long-wavelength light, such as red light, is usually unattractive to nocturnal insects [60] and might be useful for the selective trapping of some ant species.

In general, both gypsy moths and oak silkmoths were lured more frequently to UV-A lights than to lights with visible wavelengths. A more detailed examination, however, showed that the light preferences of gypsy moths were somewhat extended to longer wavelengths, including green light, whereas those of oak silkmoths were strongly biased toward the UV-A range (Fig. 5c, d). These findings are consistent with the results of studies that showed that moths with relatively large eyes and a relatively large body mass were attracted to light dominated by shorter wavelengths [48, 63, 67], although the degree of attraction varied between moth taxa [46]. One might speculate that their distinct preferences are due to the distinct spectral sensitivities of the compound eye. Asian gypsy moths showed a maximum response peak in the 480–520-nm range (blue-green region) and a secondary peak in the 340–380 nm range [14, 21]. However, whether oak silkmoths exhibit the highest sensitivity in the UV range or in the green range has not been confirmed [26], and further investigation is needed.

For forecasting the emergence of adult gypsy moths, we found generally positive correlations between the catch rate and daily temperature, supporting the belief among insect collectors that “largest numbers of moths are captured on warm days” [37]. Given that both univoltine and multivoltine moths prefer permissible ranges of ambient temperatures according to light trap sampling [1, 65], temperature is likely critical for the flight activity of exothermal moths that inhabit cool-temperate and subarctic regions.

As in European gypsy moths [49], eclosion, mating, and flight activities of Asian gypsy moths are strictly governed by diel photoperiodicity. The eclosion of Asian gypsy moths as well as mate calling by females using sex pheromones occurs in the morning, and subsequent mating occurs in the afternoon [16, 49]. At dusk, mated and unmated female Asian gypsy moths almost simultaneously begin wing fanning to raise the temperature of in the flight muscles and, minutes later, engage in synchronous, mass flight [19, 39]. Similar dusk flight has been reported in *L. monacha* and *L. umbrosa* [62]. The ambient temperature could affect this stereotyped behavioral sequence. For example, it was shown that temperatures above 24 °C enhanced eclosion [49]. Therefore, the scale of mass flight greatly depends on the number of females that eclose on the same day. Whereas immediate flight is possible at temperatures above 22 °C [18, 19], preflight warming is needed at temperatures below 22 °C [17, 18]. Thus, a low temperature might be a negative factor that desynchronizes mass flight [19].

The results of our experiments conducted at different sites and in different years support this temperature-flight relationship observed in gypsy moths. The correlation with the highest temperature on the catch day was strongest at Mt. Usu in 2014, when mass flight of gypsy moths occurred. In Yubari in 2017, when the emergence of gypsy moths was sporadic, the correlation with dusk temperature at 20:00 was the strongest. This difference might be at least partly due to climatic differences at the study sites. Since the average temperature at 20:00 in August at Yubari was lower (18.5 °C) than that (21.0 °C) at Mt. Usu, the immediate factor, dusk temperature, might be a determinant of flight activity in gypsy moths and applied in control methods.

In contrast, for Japanese oak silkmoths, there were no strong correlations between daily catch and any of the weather parameters. The difference in the dependence on ambient temperature between gypsy moths and oak silkmoths might be due to differences in their physiology and flight capabilities. Oak silkmoths, which are abundant in late autumn [30], obviously adapt more easily to lower temperatures than gypsy moths. Whereas the flight capability of female gypsy moths is limited to 200 m on average per night [36] due to high wing loading (body mass per unit wing area: 75 mg/cm^2^) [19], saturniid moths can generally maintain long-term flight due to the low wing loading (20–50 mg/cm^2^) [9], suggesting that the activity of saturniid moths is less affected by immediate weather factors than that of gypsy moths. Denno and Dingle (1981) suggested that larger bodied insects in temperate summer environments are less impacted by short-term environmental changes than smaller bodied insects [24]. Results similar to ours have been reported in macromoths that inhabit temperate zones [7]; large *Actias luna* moths (male forewings: 45*–*60 mm) have better control of overheating or overcooling than do small *Dryocampa rubicunda* moths (male forewings: 17*–*29 mm). Although much is known about gypsy moths, additional studies on the flight-to-light behavior of Japanese oak silkmoths is needed in the future.

## Conclusions

The use of UV LEDs was more effective in capturing gypsy moths and oak silkmoths than was the use of commercial UV-free lights. The light preferences of gypsy moths were somewhat biased toward longer wavelengths, including green light, whereas those of oak silkmoths were strongly biased toward the UV-A range. The highest daily temperature is the most reliable index for predicting mass flights of gypsy moths, though dusk temperature might affect the mass flight of gypsy moths in inland areas.

## Supplementary Information

**Additional file 1.** A movie of the elevated expressway close to the Yubari Interchange showing abundant egg masses of gypsy moths and a female approaching the pier.

**Additional file 2.** Configurations of the light traps used at the Mt. Usu rest area in 2014 and 2015. We used a “light tower” configuration in which there was a U-shaped white tarpaulin sheet with an array of fluorescent lights placed vertically in the middle.

**Additional file 3.** Configurations of the light traps used in 2017. Six flight-interception LED traps that emitted distinct light wavelengths were used. A UV LED was introduced to an array of visible-light LEDs in the middle of the peak emergence of gypsy moths. The traps used on from 28-September 11, 2017, were reused in 2018.

**Additional file 4.** Classification of insects captured by light traps in Yubari in 2017 and 2018.

**Additional file 5.** Emission power spectrum of each light (or each LED module) and its illuminance (lx) measured at 80 cm from the light. The values represent an average of 10 consecutive measurements using a spectroradiometer (CL-500A, Konica Minolta, Japan).

**Additional file 6.** Raw data and bar graphs based on results of experiments in 2017 showing the preferences of aquatic insects including caddisflies and dobsonflies to broadband wavelengths compared to moths.

**Additional file 7.** Correlations between daily gypsy moth catch and meteorological factors at the Mt. Usu rest area in 2014, corresponding to the last year of the gypsy moth outbreak.

**Additional file 8.** Correlations between daily gypsy moth catch and meteorological factors in Yubari in 2018, corresponding to the innoxious phase of gypsy moths.

**Additional file 9.** Correlations between oak silkmoth catch and meteorological factors in Yubari in 2018.

## Data Availability

The datasets used and/or analyzed in the current study are available from the corresponding author upon reasonable request.

## Abbreviations

LED: Light emitting diode
UV: Ultraviolet
