# Supplementary figures and images for: Management of flying insects on expressways through an academic-industrial collaboration: evaluation of the effect of light wavelengths and meteorological factors on insect attraction
Source: Zoological Lett. 2020 Nov 26;6:15. doi: 10.1186/s40851-020-00163-7 (PMC7690004; doi:10.1186/s40851-020-00163-7)

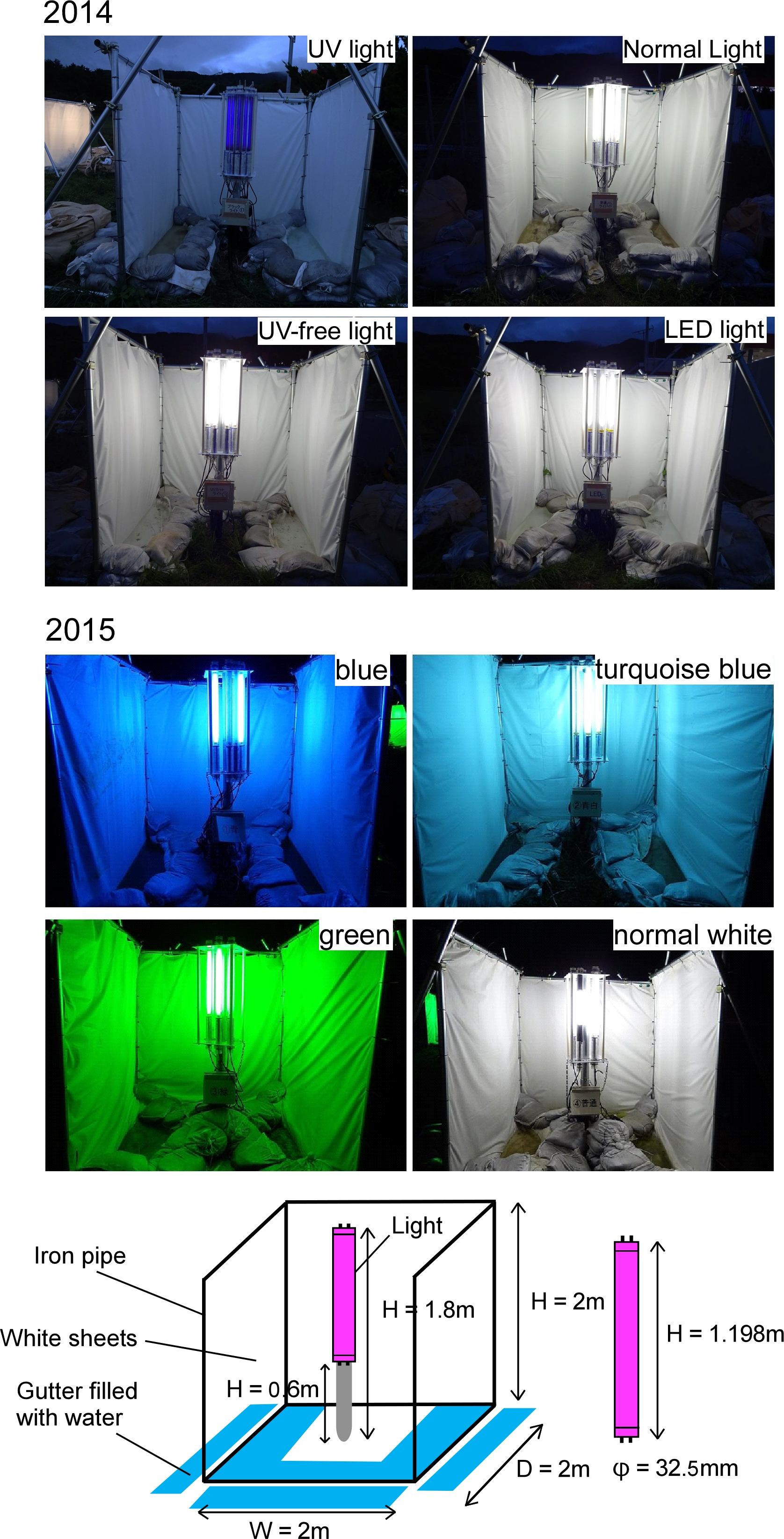

Supplement: Supplementary file 2 — Additional file 2. Configurations of the light traps used at the Mt. Usu rest area in 2014 and 2015. We used a “light tower” configuration in which there was a U-shaped white tarpaulin sheet with an array of fluorescent lights placed vertically in the middle. [file 40851_2020_163_MOESM2_ESM.jpg]

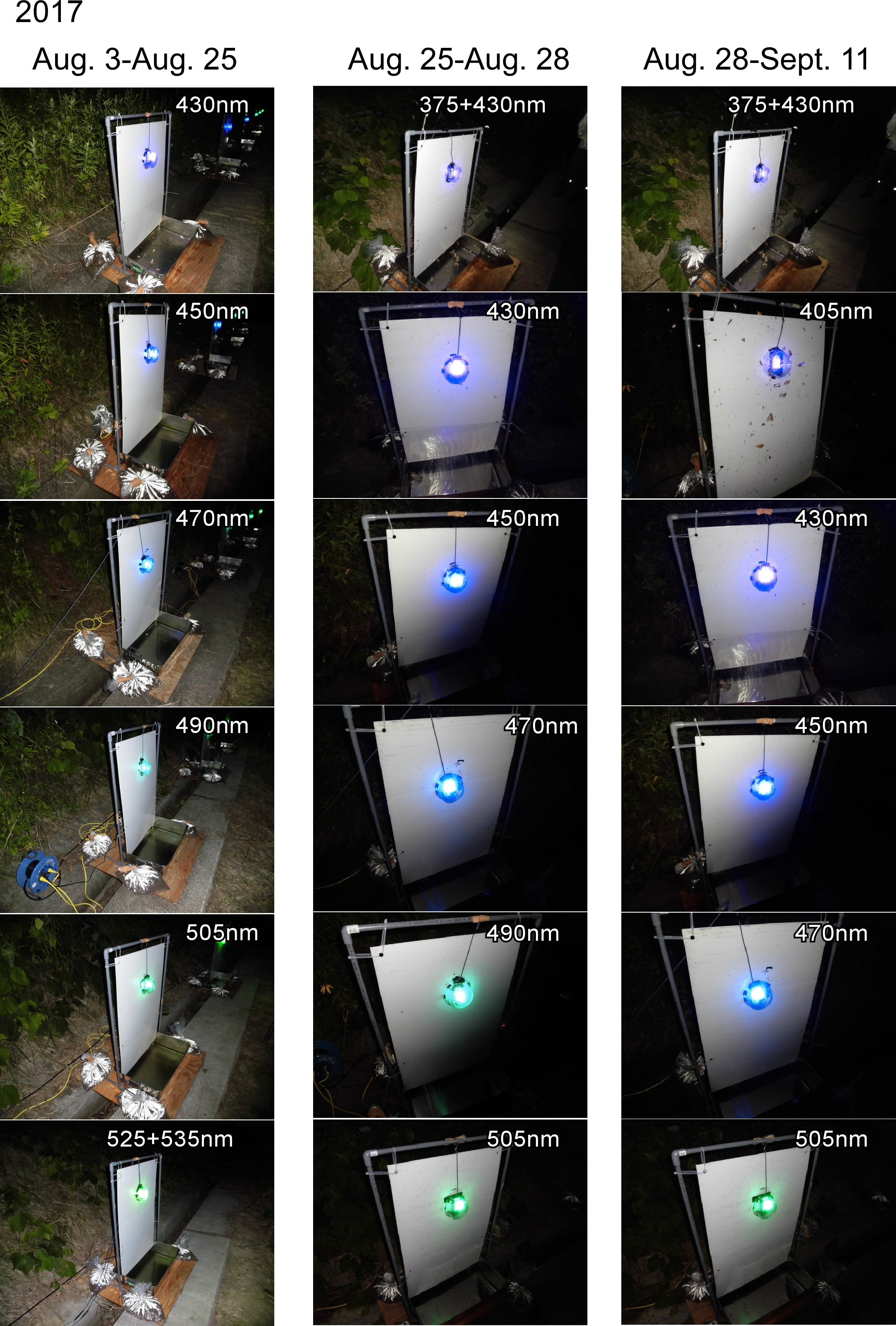

Supplement: Supplementary file 3 — Additional file 3. Configurations of the light traps used in 2017. Six flight-interception LED traps that emitted distinct light wavelengths were used. A UV LED was introduced to an array of visible-light LEDs in the middle of the peak emergence of gypsy moths. The traps used on from 28-September 11, 2017, were reused in 2018. [file 40851_2020_163_MOESM3_ESM.jpg]

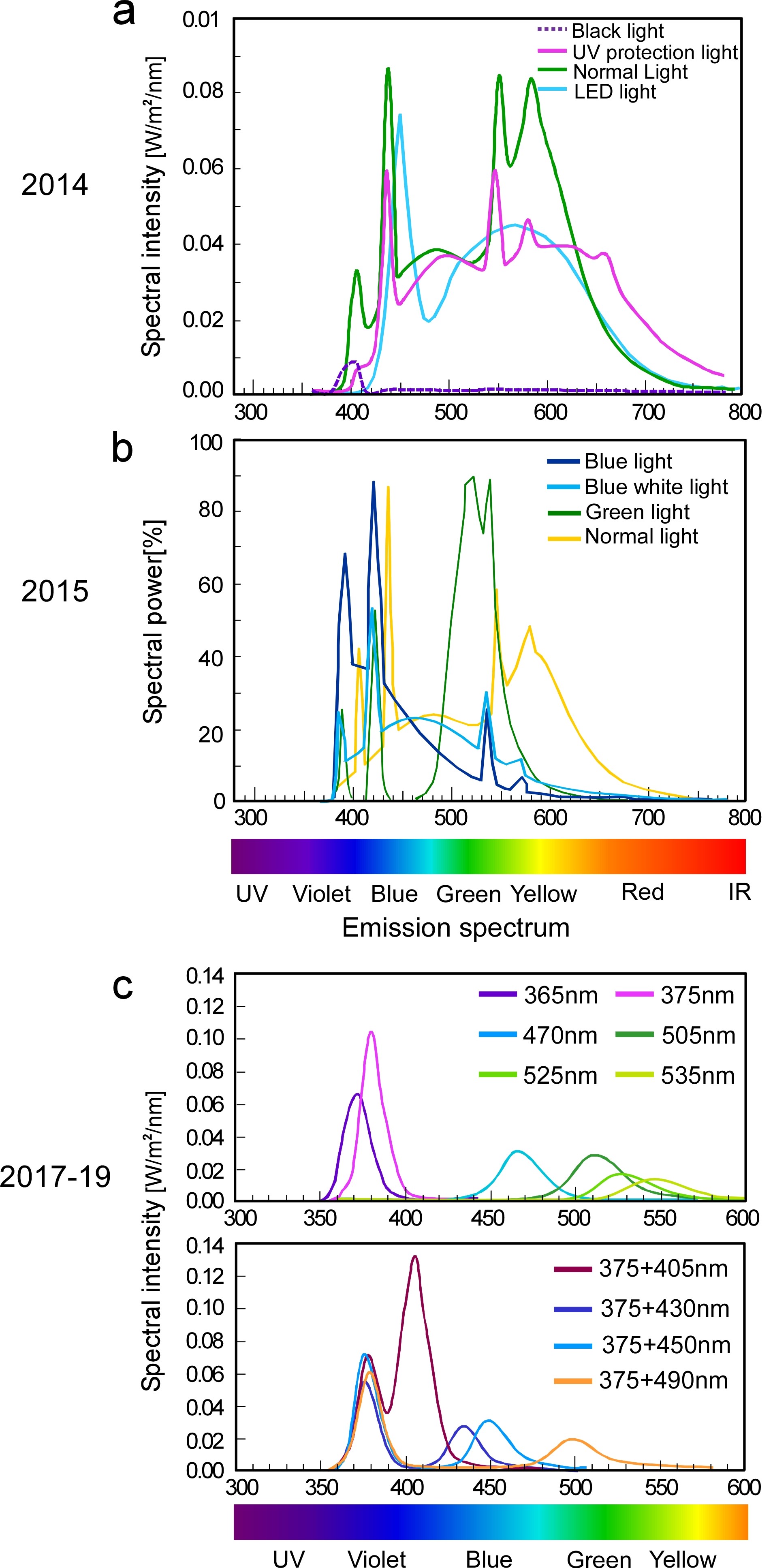

Supplement: Supplementary file 5 — Additional file 5. Emission power spectrum of each light (or each LED module) and its illuminance (lx) measured at 80 cm from the light. The values represent an average of 10 consecutive measurements using a spectroradiometer (CL-500A, Konica Minolta, Japan). [file 40851_2020_163_MOESM5_ESM.jpg]

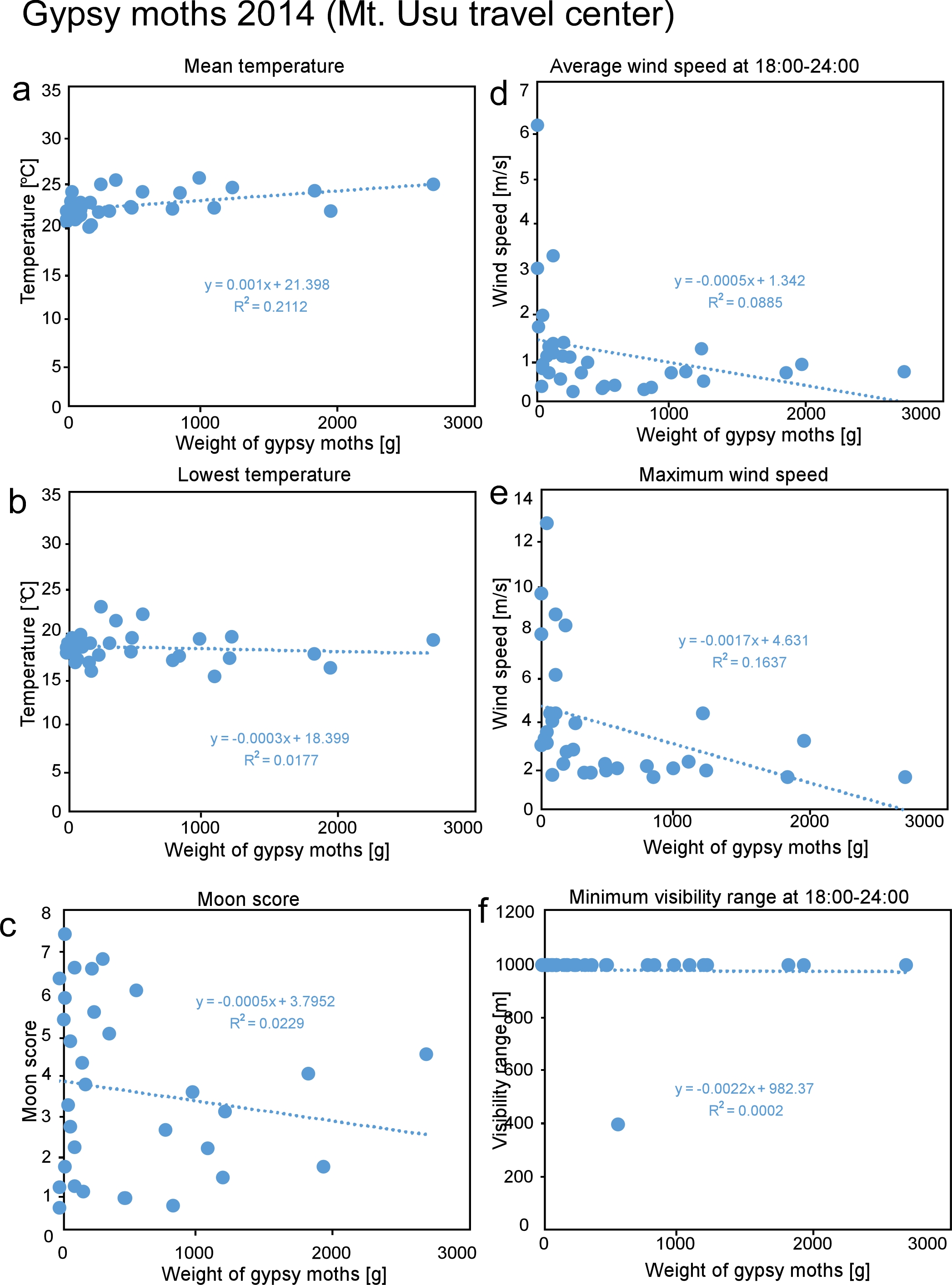

Supplement: Supplementary file 7 — Additional file 7. Correlations between daily gypsy moth catch and meteorological factors at the Mt. Usu rest area in 2014, corresponding to the last year of the gypsy moth outbreak. [file 40851_2020_163_MOESM7_ESM.jpg]

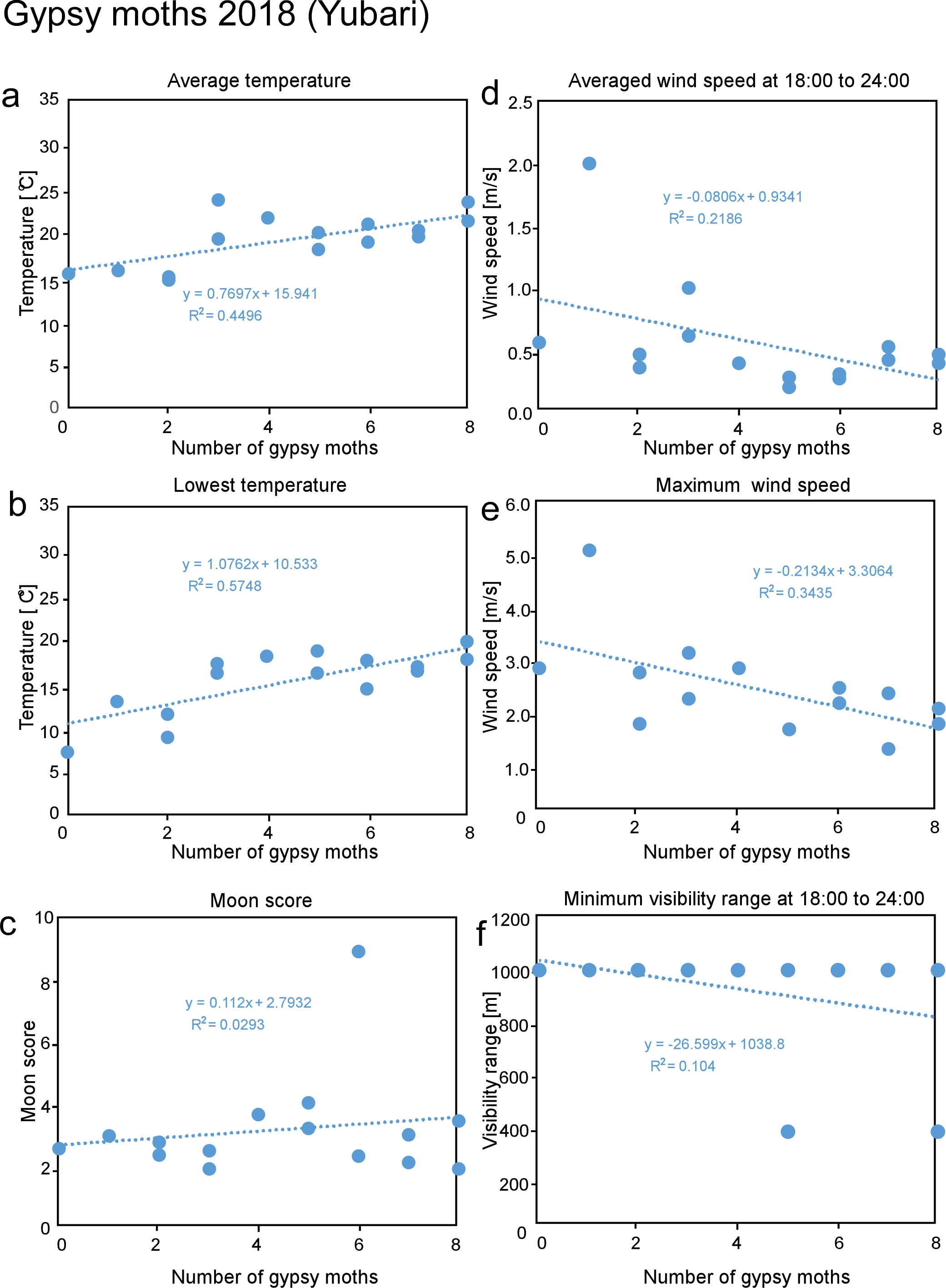

Supplement: Supplementary file 8 — Additional file 8. Correlations between daily gypsy moth catch and meteorological factors in Yubari in 2018, corresponding to the innoxious phase of gypsy moths. [file 40851_2020_163_MOESM8_ESM.jpg]

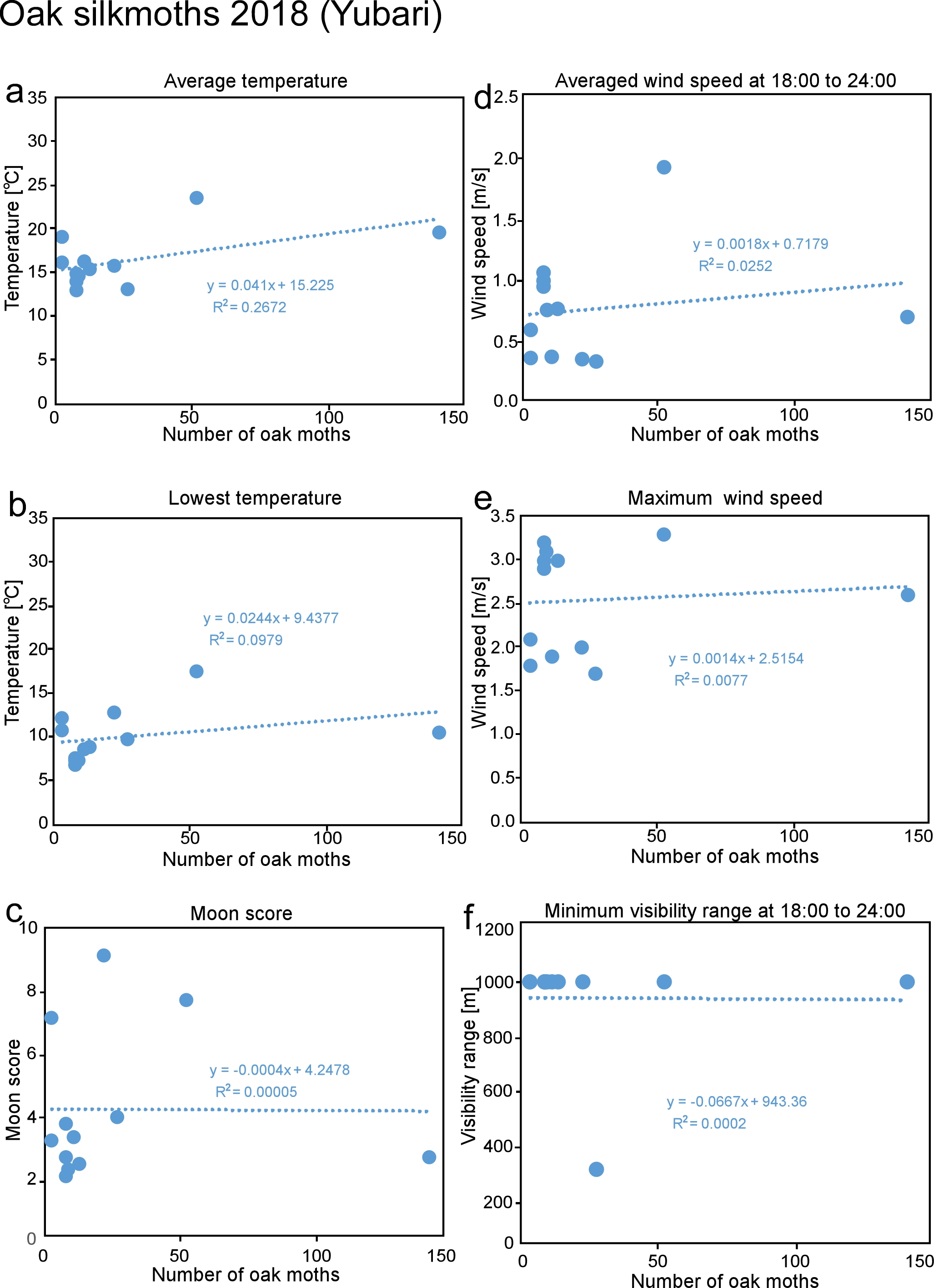

Supplement: Supplementary file 9 — Additional file 9. Correlations between oak silkmoth catch and meteorological factors in Yubari in 2018. [file 40851_2020_163_MOESM9_ESM.jpg]
